# Liver Transplant Tolerance and Its Application to the Clinic: Can We Exploit the High Dose Effect?

**DOI:** 10.1155/2013/419692

**Published:** 2013-11-06

**Authors:** Eithne C. Cunningham, Alexandra F. Sharland, G. Alex Bishop

**Affiliations:** Collaborative Transplantation Research Group, Bosch Institute, Royal Prince Alfred Hospital and University of Sydney, Sydney, NSW 2006, Australia

## Abstract

The tolerogenic properties of the liver have long been recognised, especially in regard to transplantation. Spontaneous acceptance of liver grafts occurs in a number of experimental models and also in a proportion of clinical transplant recipients. Liver graft acceptance results from donor antigen-specific tolerance, demonstrated by the extension of tolerance to other grafts of donor origin. A number of factors have been proposed to be involved in liver transplant tolerance induction, including the release of soluble major histocompatibility (MHC) molecules from the liver, its complement of immunosuppressive donor leucocytes, and the ability of hepatocytes to directly interact with and destroy antigen-specific T cells. The large tissue mass of the liver has also been suggested to act as a cytokine sink, with the potential to exhaust the immune response. In this review, we outline the growing body of evidence, from experimental models and clinical transplantation, which supports a role for large tissue mass and high antigen dose in the induction of tolerance. We also discuss a novel gene therapy approach to exploit this dose effect and induce antigen-specific tolerance robust enough to overcome a primed T cell memory response.

## 1. Liver Transplant Tolerance

From the very first experimental liver transplants, it was clear that livers were less likely to be rejected than other transplanted organs [1]. In animal models, liver transplants are often accepted without requiring any treatment while other transplanted organs, such as hearts or kidneys, are rejected. This was first demonstrated in outbred pigs [1] and subsequently in inbred rats [2, 3] and mice [4].

Spontaneous acceptance of a transplanted liver leads rapidly to liver donor-specific tolerance in many models [1, 2, 5, 6]. This tolerance is particularly robust and rapidly induces acceptance of skin grafts from the liver donor strain [1, 5, 7, 8]. Moreover, a liver transplant can act like an immunosuppressive drug in reversing ongoing rejection of heart [9] or pancreas [10] transplants that are syngeneic with the liver donor. Clinical liver transplants also have a better outcome than transplants of other organs with a significant proportion of patients able to be removed from all immunosuppression [11, 12].

There have been many proposed mechanisms for the ability of the transplanted liver to be accepted by the recipient. Initially, it was thought that the high levels of soluble major histocompatibility (MHC) molecules produced by the donor liver were responsible for liver tolerance [13]. This has not subsequently been validated and it appears that soluble MHC is at best a minor component of the liver tolerance effect [14, 15]. Notably, liver transplants from animals that lack MHC molecules are not rejected [16]. Subsequently it was shown that donor passenger leucocytes play a role in liver transplant acceptance as their depletion by irradiation of the donor [17] or by parking the liver in a recipient strain animal [18] led to graft rejection. Administration of donor leucocytes at the time of transplantation of a heart [19] or kidney [20] yielded considerable prolongation of survival in animal models. However, attempts to translate these findings to a clinical setting providing leucocytes, in the form of donor bone marrow, infused at the time of transplantation have shown only very modest improvement in outcomes [21, 22].

A recently proposed mechanism for the ability of the liver to induce tolerance is based on the unique vascular architecture of the liver which allows intimate contact of circulating T cells with hepatocytes. This is facilitated by the fenestrated endothelium of the liver sinusoids, where small endothelial pores (fenestrae) permit contact between recirculating T cells and hepatocytes. The intact lining and tight junctions of the endothelium in other organs prevents contact with parenchymal cells. The slow rate of blood flow in liver sinusoids further aids the establishment of contact between circulating CD8^+^ T cells and hepatocytes [23]. Contact of T cells with hepatocytes leads to their engulfment by hepatocytes and degradation by a process termed “suicidal emperipolesis” [24] or to their abortive activation and death [25]. Both of these processes lead to clonal deletion of liver-reactive T cells, a process that has been demonstrated to be responsible for liver transplant tolerance in animal models [26–29]. Despite these interesting findings, the process of suicidal emperipolesis has not yet been demonstrated in transplanted livers and its role in clinical liver transplantation has yet to be established.

A further basis of liver transplant acceptance is the large size of the liver, approximately 10 times greater than that of a heart or a kidney. This mass of tissue can function as a cytokine sink and/or dilute the finite clones of alloreactive T cells and thus potentially exhaust the recipient's immune response. There is mounting experimental evidence that the volume of allogeneic tissue transplanted is an important contributor to tolerance, as increasing the mass of transplanted tissue prolongs survival. Of considerable interest, in clinical transplantation, there is convincing evidence from many studies that multiple organ transplants from the same donor to a single recipient have a better outcome than single organs alone. As there has been no previous review of the dose effect in organ transplantation, the following sections will examine the experimental and clinical evidence for high dose tolerance and describe a gene therapy approach that can exploit it as a potential means to induce antigen-specific tolerance.

## 2. High Dose Tolerance in Transplantation 

### 2.1. Dose Effects in Animal Models of Transplantation

The first description of a dose effect in transplantation was Medawar's landmark study on the immunological basis of skin graft rejection, part of which described more rapid rejection of larger compared to smaller skin grafts in rabbits [30]. In this study, the grafts were quite small (the larger grafts were approximately 8 mm in diameter and the smaller were 2-3 mm) in relation to the size of the rabbit. This finding was later confirmed, although again, the grafts were relatively small [31–33]. The opposite was observed when skin grafts were increased considerably in size in proportion to the size of the animal so that greater than approximately twenty percent of the total skin area was transplanted [32, 34–37]. These early studies of skin graft rejection indicated that very small grafts were more slowly rejected than moderate size grafts but that markedly increasing the size of the skin graft prolonged survival. 

Subsequent studies in mouse skin transplant models with minor antigen mismatches gave rise to similar findings. The first of these showed that if the donor and recipient were incompatible at loci other than H-2, larger grafts demonstrated prolonged survival. They also observed that while small secondary grafts underwent accelerated rejection, larger secondary grafts did not [38]. Similarly, a mouse model of minor antigen mismatch (i.e., the male HY antigen) showed that female mice rejected primary syngeneic male skin grafts but accepted larger grafts and female mice that had been primed by injection of male spleen cells in the footpad rejected the majority of the male grafts, regardless of the size. Interestingly, female recipients that had rejected a small, primary male graft went on to accept larger, secondary male grafts in the majority of cases [39], suggesting that a graft of sufficient size could even overcome preexisting immunity against the donor. 

Since then, studies in the fully histoincompatible rat transplant model of PVG donor to DA recipient have shown that increasing antigen load, by transplanting multiple organs, increases allograft survival rates. Transplantation of one heart or kidney in this model led to rejection in 9 and 8.5 days, respectively. Administration of donor leucocytes alone could not increase survival of a cardiac graft, but transplanting two hearts and two kidneys, with donor leucocytes, led to spontaneous acceptance and indefinite survival of the grafts [40]. Two or three hearts survived for 15.5 days, and two or three kidneys survived for 60 days and >100 days, respectively, while two hearts plus one or two kidneys prolonged their survival to >100 days [41, 42].

This dose effect was also observed in an inbred miniature swine model where single MHC class I mismatched heart allografts were rejected within 55 days after transplanting into cyclosporine-treated recipients [43]. In contrast, hearts grafted into cyclosporine-treated recipients that also received a kidney from the same donor developed rapid and stable tolerance that resulted in long-term survival of the heart [44]. Cell-mediated cytotoxicity and alloantibody production were suppressed in combined recipients and there was no evidence of cardiac allograft vasculopathy. To address if this effect was specific to the kidney, the authors transplanted 2 hearts, MHC matched to each other, but class I mismatched to the host. These recipients also displayed significantly prolonged (>190 days) cardiac allograft survival [45]. Conversely, when the mass of an organ that would usually be accepted is reduced, graft survival declines. In experimental porcine liver transplants, rejection was more frequently observed in small accessory livers than large orthotopic livers [1].

**Figure 1:**
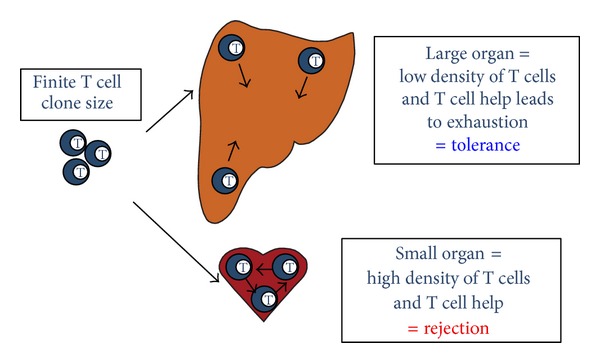
High antigen dose leads to tolerance by exhausting the finite T cell clone size. Transplantation of a smaller organ (e.g., a heart) results in rejection due to a high density of alloreactive T cells and sufficient T cell help. Grafting a large tissue mass or organ (e.g., a liver) leads to a low density of alloreactive T cells and T cell help, resulting in exhaustion of T cells and subsequent tolerance.

One possible explanation for the prolonged survival of very large grafts is nonspecific immunosuppression due to the increased trauma associated with the surgery involved in transplanting massive or multiple grafts [34]. Alternatively, survival prolongation could be due to the increased mass of tissue transplanted, which exhausts the recipient's immune response. One mechanism for this exhaustion could be that there is a limited clone size of graft-reactive T cells which are unable to establish “critical mass” (Figure 1). This is analogous to a nuclear chain reaction, where fission does not occur until there is sufficient density of free neutrons. In transplantation, rejection might necessitate a sufficient density of activated high-affinity T cells, which is more difficult to achieve with a limited T cell clone size in a larger mass of tissue.

Over the years, a number of rodent studies have demonstrated that there is a hierarchy of susceptibility between different graft types and sizes with skin and intestine being the most prone to rejection while islets, hearts, kidneys, and livers are progressively more easy to tolerize [46–49]. It had been suggested that this was due to the expression of tissue-specific antigens on grafts but development of a mouse model allowing for examination of the response to different grafts without needing to consider tissue specific antigens showed that this was not the case [50]. In this model, T cells were depleted from the recipients, which were then reconstituted with a specific subset of T cells reactive against one MHC antigen, H-2K^b^, expressed on the donor grafts. T cell-depleted mice were immunocompromised and were unable to reject H-2K^b^-expressing heart, skin, or islet grafts without the addition of the alloreactive T cells. Adoptive transfer of the H-2K^b^-specific T cells resulted in rejection of all three types of graft although many more cells (~6000-fold) were required to mediate rejection of the heart grafts than the skin and islet transplants, confirming that the latter two are more susceptible to rejection. This suggested that a threshold number of donor-reactive T cells is required for graft rejection and that this threshold is higher in heart grafts compared to skin or islets.

These findings were confirmed in a polyclonal system by a study which assessed the T cell response following heart and skin grafting in a mouse model of HY minor antigen mismatch [51]. Female mice rejected male skin grafts but accepted male hearts indefinitely, as expected. This was despite the fact that the frequency of donor-reactive T cells specific for male antigens and the cytotoxic activity and cytokine profile of the cells was similar in recipients of skin and heart grafts. The authors reported that the heart transplant primed a proinflammatory, anti-male T cell immune response, but that it was insufficient in quantity to mediate acute rejection of the graft. They used a TCR-transgenic to show that the male grafts do express enough antigen to be rejected if sufficient numbers of high-affinity T cells are present. The investigators also addressed the issue of graft size by transplanting female recipients with cardiac allografts from young, and, therefore, small males. The majority of these heart grafts, which were 50% of the weight of normal adult donor hearts, were acutely rejected within 25 days. The frequency of alloreactive T cells was the same, supporting the idea that the ability of a defined number of effector T cells to reject a transplanted organ depends on the size of the graft. Furthermore, while female recipients acutely rejected one male skin graft, survival of two grafts was markedly prolonged, and the number of antidonor T cells in both recipients was the same. Overall, these studies confirmed that a threshold number of cells seemed to be required for graft rejection and they suggested that larger grafts might be rendered resistant to rejection by exhausting the immune response. 

A more recent study used the model described above [50], in which T cell-depleted recipients were reconstituted with H-2K^b^-specific T cells, to examine the response in mice following transplantation of H-2K^b^-expressing heart, kidney, and liver grafts [29]. Transfer of the same number of alloreactive T cells resulted in acceptance of the liver grafts but rejection of the kidney and heart allografts. They found that most of the alloreactive T cells had proliferated and differentiated into memory or effector cells after liver transplantation and were detected in the lymphoid tissues and the liver allograft. Some activation and proliferation were seen after kidney and heart transplantation, but naive alloreactive cells remained in the lymphoid tissues, long term. The authors concluded that following transplantation of a liver graft, the rapid and extensive T cell activation resulted in their clonal exhaustion or deletion. 

Some recent work has examined whether this tolerance is due to exhaustion by examining the expression of various *γ* chain cytokines and their receptors in the PVG to DA rat model of transplantation [52]. In this model, heart and kidney transplants are rejected in <10 days while liver transplants are accepted for >100 days. Cytokine levels (IL-2, IL-4, IL-7, IL- 9, IL-15, and IL-21) and their receptors (*γ*c, IL-2R*α*, IL-2R*β*/IL-5R*β*, IL-4R*α*, IL-7R*α*, IL-9R*α*, IL-15R*α*, and IL-21R*α*) were assessed by qPCR at days 3, 5, and 7 following grafting and, except for IL-21, the levels of the *γ* chain cytokines and receptors were lower in transplanted livers than in hearts or kidney. As *γ* chain-signalling is crucial for T cell survival, this indicated that the tolerance seen in this model may be due to a low level of signalling, reflecting the “dilution” of a fixed number of alloreactive T cells in a large organ or tissue mass. 

### 2.2. Evidence of the Dose Effect from Clinical Transplantation

The observations made in animal models relating to the tolerogenicity of the liver have also been supported by clinical transplantation data. There have been a number of anecdotal reports of tolerant patients, resulting from a variety of treatments, and up to 15% of liver transplant recipients have been shown to be able to completely discontinue immunosuppressive therapy (see [11, 12] and reviewed in [53]). In addition, ranges of clinical studies, analysing the survival of various transplanted organs, have confirmed the ability of simultaneous transplantation to protect organs from rejection.

The first studies on combined liver-kidney transplants, on small groups of seven patients reported little or no [54, 55] evidence of renal graft rejection (much less frequent than in patients receiving a kidney alone). This has been overwhelmingly confirmed over the years (despite some reports to the contrary [56], which may not have taken into account the immunological status of the recipients) by a number of single-centre studies [57–59] and by more large scale analysis [60, 61], including a single-centre study and analysis of UNOS data on kidney survival in infants and children [62]. A recent analysis of UNOS data from 1996 to 2003, of 1,136 combined liver-kidney transplant recipients and 352 patients receiving liver transplants, followed by kidney grafts from different donors, confirmed that the protective effect of the liver was donor specific [63]. There are also a number of reports of a combined transplant enabling successful kidney grafts, despite a positive crossmatch between donor and recipient, which usually results in hyperacute rejection of the kidney [64–67]. One study found that even a partial auxiliary liver transplant from the kidney donor can protect the kidney in a positive cross-match situation [68]. Some of these studies have also reported a lower rate of liver rejection in patients after combined liver-kidney transplantation, compared to those receiving livers alone [55, 58]. 

There is also a growing body of evidence that this effect is not liver-specific but is instead related to the antigen load. As with animal models, it has been observed that larger organs can fare better after transplantation. For example, the report mentioned above, which examined the survival of kidneys in infant and child recipients, demonstrated a substantial protective effect of using larger, adult-size kidneys compared to smaller grafts. The authors suggested that the exposure of lymphocytes to a large dose of antigen may be leading to exhaustion [62]. An analysis of data provided to the international Collaborative Transplant Study on the survival of kidneys transplanted alone, compared to combined transplants, showed that the survival was equally high in patients receiving combined heart-kidney grafts, as in those receiving liver-kidney grafts. However, they could not rule out the possibility that the improved survival in heart-kidney graft recipients was due to a higher dose of immunosuppression [60]. Similarly, the first multiinstitutional study, of 29 centres in the United States, found that 82 recipients transplanted with a combined heart-kidney transplant had lower incidences of cardiac rejection than those transplanted with a heart alone. The authors hypothesised that it might be related to antigen load, although again they also could not completely rule out the possibility that double organ recipients might have less rejection due to increased immunosuppression [69]. There are now several subsequent reports of lower rates of graft rejection in recipients of combined heart-kidney transplants. A number of other single-centre studies have confirmed that the incidence of cardiac rejection is much lower in recipients receiving combined heart-kidney grafts compared to hearts alone, [70–74]. All of these studies (except the first which did not assess kidney graft survival) also reported little or no renal graft rejection, despite a lack of HLA matching. More recently, a large study of 67 patients in three French centres, receiving combined heart-kidney transplants between 1984 and 2007, also found very low rates of cardiac and renal allograft rejection [75].

One of the most comprehensive analyses of the effect of combined organ transplantation to date looked at rejection rates in UNOS data for a total of 133,416 allograft recipients [76]. They found that heart, kidney, and liver grafts were protected and were able to protect each other, with lower rates of rejection and greater rejection-free survival of grafts apparent in the setting of combined transplantation. Specifically, the authors reported a lower rate of liver rejection in liver-kidney recipients, compared to patients receiving livers alone. There was also a reduced rate of kidney graft rejection in recipients of both heart-kidney and kidney-liver transplants, compared to kidneys transplanted alone, although they acknowledged a possible contribution of higher immunosuppression therapy to the reduction of the former. However, differences in immunosuppressive therapy were not responsible for the reduction in cardiac graft rejection seen in both heart-kidney and heart-liver transplants, when compared to heart transplants alone. The analyses also showed that recipients of double-lung and double-kidney transplants both had less rejection and improved rejection-free survival compared to single transplants, providing evidence that antigen load is an important factor. Previously, it has been suggested that combined pancreas-kidney transplants led to a reduction in pancreas allograft rejection [77]. This was not supported by this report, although the data for pancreas were more difficult to interpret, due to significant differences between groups in age and disease state. Similarly, there was a suggestion from this study that intestinal grafts had reduced rejection when transplanted with a liver, but differences in patient groups also complicated the data in this case [76].

A recent large-scale analysis of multiorgan transplantation in patients over a 12-year period at the University of Alabama, Birmingham, AL, USA examined the effect of combined heart-kidney and heart-lung transplants [78]. In general, they found that the probability of acute rejection and the number of rejection episodes were much lower in recipients of more than one organ from the same donor. As seen in other studies, they reported significantly reduced cardiac rejection in heart-kidney recipients compared to that seen in patients receiving a cardiac graft alone. They also reported a significant reduction in cardiac rejection episodes in combined heart-lung recipients, confirming previous observations [79–81]. They also saw reduced lung rejection in double-lung recipients compared to heart-lung transplant recipients [78]. 

More recently, in clinical composite tissue transplantation, where skin is transplanted as a component of a much larger tissue mass, skin survival is enhanced [82] compared to that of skin transplanted alone [83], supporting older anecdotal evidence that large experimental skin grafts in human burns patients survived considerably longer than small grafts [32, 34]. Overall, it is becoming clearer from these smaller studies and large-scale analyses that reductions in rejection rates are not only associated with liver tolerogenicity, but may be related to the antigen load of organs transplanted instead.

## 3. Gene Therapy Approach in Mice Can Exploit the Antigen Dose Effect

The apparent importance of antigen dose in liver tolerance led us to use a liver-directed gene therapy approach to attempt to exploit this in a mouse skin transplant model [84]. Previously, induction of tolerance to a foreign protein has been shown to be facilitated by expression in the liver (reviewed in [85]). In particular, it has been found that hepatocyte-restricted antigen expression, with no expression in professional antigen presenting cells [86], and higher levels of gene expression (reviewed in [87]) are important. In one case, targeting the expression of a neural autoantigen to the liver was able to induce tolerance to subsequent neural autoimmunity in a mouse model of multiple sclerosis [88].

We employed a minimally immunogenic recombinant adenoassociated virus (rAAV) vector, designed to specifically express high levels of antigen in host livers [89, 90] to assess tolerance induction in a mouse skin transplant model. We examined whether use of this system to express high levels of the mouse major histocompatibility locus (MHC) antigen H-2K^b^ in the recipient liver could induce long-term acceptance of skin grafts expressing this antigen. B10.BR (MHC k haplotype) mice were injected with rAAV-H-2K^b^ to induce specific expression of H-2K^b^ on their hepatocytes [84]. These recipients were grafted after 7 days with skin from 178.3 mice that transgenically express H-2K^b^ on a k haplotype background. Therefore, we were able to express the single, mismatched MHC antigen, found on the donor skin graft, in the recipient's liver prior to transplantation. Uninjected B10.BR mice rejected their 178.3 skin grafts with an MST of 18.5 days, while mice injected with rAAV-H-2K^b^ accepted their grafts long term. Injection with low doses of rAAV-H-2K^b^ did not produce the same result. This confirmed that high-level, liver-directed donor MHC antigen expression was able to promote tolerance to donor-strain skin grafts.

We were able to achieve this tolerance even in animals previously primed to produce a memory response to the antigen. Mice were primed with an H-2K^b^-expressing skin graft which they rejected in the expected time. Uninjected mice rejected secondary 178.3 skin grafts with an accelerated tempo, as expected. However, primed mice that were then treated with rAAV-H-2K^b^ accepted their second skin grafts in the majority of cases, reminiscent of the effect observed previously where skin grafts onto rats that had received a liver transplant from the same donor survived for long term [5]. This was of particular interest for the clinical setting, as any potentially useful gene therapy system would need to be able to overcome the possible barrier of preexisting immunity in patients due to heterologous immunity [91]. We found that injection of mice, previously primed with a 178.3 skin graft, with rAAV-H-2K^b^ led to a significant reduction in the number of cells expressing IFN*γ* in response to H-2K^b^. However, we did not see deletion of H-2K^b^-reactive cells and we were also unable to demonstrate any linked epitope suppression following gene transfer, which might have indicated development of regulatory cells. Therefore, we suggest that the mechanism of tolerance induction in this case is a form of functional silencing of alloreactive T cells, mediated by high-level expression of antigen in hepatocytes.

Expression of donor MHC, following gene transfer to different recipient cell types in rodents, has previously produced variable results regarding allograft survival. A study of the ability of MHC class I or II expression to induce unresponsiveness to cardiac allografts in mice showed that pretreating recipients with cells (expressing a variety of MHC antigens) before grafting was able to prolong survival. However, this was dependent on the immunogenicity of the antigen and the load delivered [92]. Similarly, retroviral transduction of bone marrow cells with donor MHC antigen led to prolongation of skin grafts [93] in mice. However, gene transfer of donor MHC to skeletal muscle in a rat model resulted in accelerated rejection of cardiac grafts [94], but it is possible that the dose of antigen was not sufficient to induce tolerance in this case. It has been observed that lower doses of rAAV vector targeted to the muscle can stimulate an antibody response while higher doses did not result in antibody production [95, 96]. These vectors were also targeted to muscle rather than liver and it would be interesting to ascertain whether expression in muscle is as effective for tolerance induction as expression in liver. 

## 4. Conclusions

We have previously suggested a mechanism of tolerance based on the idea that there is a relationship between antigen dose and tolerance induction [97]. At low doses of antigen or stimulation, there is immunological ignorance, and as the dose or stimulation increases, there is a corresponding increase in the immunological response, resulting in rejection of allografts when a certain threshold is reached. At very high doses of antigen, there is too much stimulation, and there is a corresponding exhaustion of the immune response, leading to tolerance induction and acceptance of allografts. A similar model of immune reactivity (the antigen-localization-dose-time and structure model) has also been described elsewhere [98].

Overall, the evidence from experimental and clinical transplantation seems to support this type of model. Initially, it seemed that the liver had a unique ability to be accepted and could protect other organs against rejection. However, following the confirmation, from both animal models and clinical analyses, that other organs and multiple organs can exert a similar affect, it seems more likely that antigen dose plays a larger role in this process than simply the type of graft. From our work with liver-directed expression of donor MHC mediated by the AAV vector system, we have been able to confirm that protection to other organs can be induced solely by expression of high doses of donor antigen in the liver. This work has not delineated the relative contributions of liver-specific expression and high-level expression to tolerance induction. Further studies using this approach to achieve high-level expression of donor MHC in nonliver tissues should yield this information.
